# The Effect of Short-Term Wingate-Based High Intensity Interval Training on Anaerobic Power and Isokinetic Muscle Function in Adolescent Badminton Players

**DOI:** 10.3390/children8060458

**Published:** 2021-05-31

**Authors:** Duk-Han Ko, Yong-Chul Choi, Dong-Soo Lee

**Affiliations:** 1 Department of Sports Science Convergence, Dongguk University, Seoul 04620, Korea; kodh119@hanmail.net; 2 Department of Physical Education, Gangneung-Wonju National University, Gangneung 25457, Korea; skicyc@gwnu.ac.kr; 3 Lee Dong Soo Badminton Academy, Seoul 06548, Korea

**Keywords:** badminton, short-term training, high-intensity interval training, adolescent anaerobic

## Abstract

Badminton requires both aerobic fitness and anaerobic ability for high performance. High intensity interval training (HIIT) is a traditional training method for improving fitness. In this study, we investigated whether short-term Wingate-based HIIT is effective for improving anaerobic activity in youth badminton players. Participants included 32 total badminton players in middle school and high school. They were divided into two groups (HIIT and moderate continuous training (MCT)). Training occurred for 4 weeks in total, three times a week, for 30 min each session. A body composition test, isokinetic knee muscle function test (60°/s, 240°/s), Wingate anaerobic power test (30 s × 5 sets), and analysis of heart rate changes were undertaken before and after training. After 4 weeks, body fat decreased in the HIIT group (*p* = 0.019); they also showed superior anaerobic ability compared to the MCT group. Differences were statistically significant in 3–4 sets (three sets, *p* = 0.019; four sets, *p* = 0.021). Regarding fatigue, the HIIT group showed superior fatigue improvement after training and better fatigue recovery ability in 3~5 sets (three sets, *p* = 0.032; four sets, *p* = 0.017; five sets, *p* = 0.003) than the MCT group. Neither group exhibited changes in heart rate during the anaerobic power test after training. Both groups improved in terms of isokinetic knee muscle function at 60°/s with no differences. However, at 240°/s, the HIIT group showed a statistically significant improvement (*p* = 0.035). Therefore, HIIT for 4 weeks improved the athletes’ performance and physical strength.

## 1. Introduction

Badminton is a very popular sport globally. According to the International Olympic Committee (IOC) and the World Badminton Federation, more than 200 million people in the world enjoy this sport. Badminton requires the high-intensity use of the joints for various actions that take place in a short time in a rectangular court [1]. It also requires a high level of physical fitness, as athletes are given only short breaks between matches [2]. The physical factors that affect badminton performance include not only cardiorespiratory endurance, agility, and quickness, but also anaerobic power [3]. In particular, repetitive jumps, continuous rallying, and the agility necessary for changes in movement direction are required in order to traverse large areas of the court very quickly [4]. Therefore, for badminton players, VO2 max is an important factor in determining athletes’ performance [5]. In addition, anaerobic ability is required in badminton to perform the necessary intermittent, rapid, and accurate movements while maintaining balance [6]. Sports drills that require frequent long steps after repeated smashing or stopping may offer better performance for athletes with higher functional capacity [7]. In badminton games, about 60–70% of the game uses an aerobic energy substrate, leaving about 30–40% using an anaerobic energy substrate [4]. The aerobic energy substrate refers to the process of synthesizing ATP by introducing the ingested nutrients into the mitochondria, and the anaerobic energy substrate refers to a state that does not require oxygen to generate energy. Previously, various training methods have been applied to improve anaerobic power. One of these methods is high-intensity interval training (HIIT). HIIT is an interval-based form of exercise that maintains more than 80% of the maximum oxygen intake. In many reports, HIIT has been found to be the best way to improve aerobic and anaerobic capacity. Its benefits also appear more quickly than those of traditional training methods [8,9]. Therefore, HIIT has been commonly used for the training of athletes since the 1950s [10]. It is designed to increase the lactate and anaerobic thresholds through repeating periods of high intensity and rest [11]. HIIT has been used by many athletes to develop their ability to recover quickly during short breaks [12]. Training has been reported to be effective when it is conducted for 8–12 weeks, and some studies have indicated that there was a significant improvement with 2–6-week periods of HIIT [13,14]. Despite its positive effects, HIIT has a high risk of adverse effects and dropout in children and adolescents with low fitness levels because it repeats and sustains high-intensity bouts of 80% or more of the maximum oxygen intake [15]. As a result of meta-analysis on dropout of HIIT in previous studies, consisting of an analysis of 1318 people in total, HIIT dropout accounted for 17.6% of participants. In that study, the factors affecting dropout were training duration (4 weeks or more), training method (running), and training intensity. In addition, the HIIT method with the lowest drop-out in that study was a cycling-based intervention [16]. In addition, many preceding studies have considered the characteristics of badminton, and then selected training methods such as agility training, stepping, and full-time running to improve performance. However, this type of training cannot accurately measure exercise intensity because the heart rate response may differ from athlete to athlete even with the same training, causing the intensity of the exercise to be relative to the participant. Therefore, it is thought that training using a stationary bicycle is conducive to observing the effect of training according to absolute exercise intensity [16]. In the end, for effective HIIT, it is necessary to study the application of the exercise period or the exercise method and its effect. Therefore, in this study, we attempted to analyze the results of applying an HIIT program to adolescent badminton players for 4 weeks. For this, the HIIT performed was centered on Wingate-based training to improve anaerobic power, and routine moderate continuous training (MCT) was performed to improve muscle strength and endurance, and this was compared to HIIT. MCT is a training method traditionally applied to athletes and the public based on continuous medium-intensity exercise [17].

## 2. Materials and Methods

### 2.1. Participants

**Figure 1:**
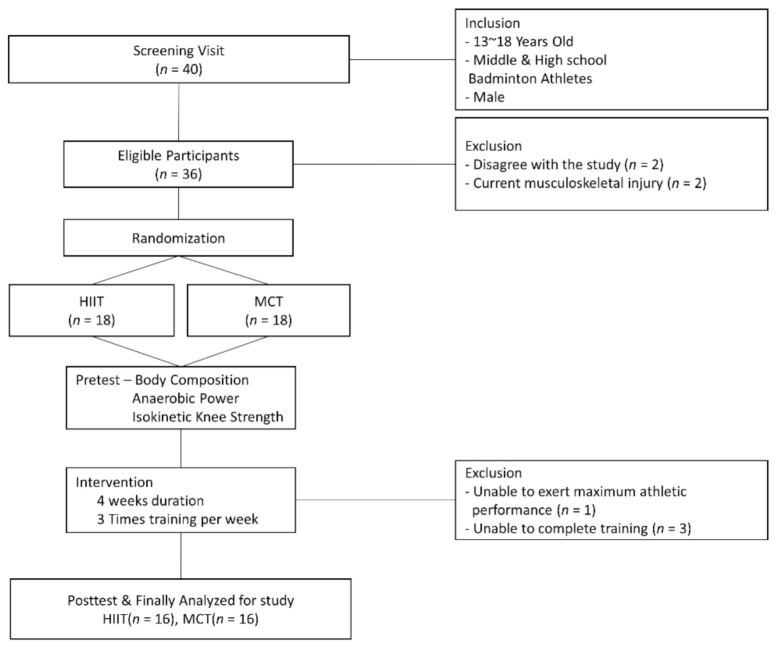
The design of Study; HIIT, high-intensity interval training; MCT, moderate continuous training.

Participants were youth badminton players who visited an athlete training center. Initially, 20 middle school students and 20 high school students were recruited, but only 15 males aged 13–15 and 17 males aged 16–18 were included in this study. Excluded subjects were 5 middle school students and 3 high school students who were currently injured (*n* = 2), did not agree to participate in the study (*n* = 2), or did not complete the experiment during training (*n* = 4). Female students were excluded because there may be gender differences in the effectiveness of training. The final 32 participants did not state that they had chronic pain in the back, knees, and ankles in a questionnaire, and did not have problems with running, jumping, and cutting movements. Athletes checked the availability and safety of the program and athletes and staff were informed of the use of the training and test results for research purposes. Those who provided written consent then completed baseline testing. In a visit to the center, body composition and physical examinations were tested, followed by the Wingate anaerobic power test and an isokinetic test. The 32 participants were randomly divided into two groups: HIIT (the intervention group) and MCT (the control group), with 16 members each. A schematic diagram of the study is presented in Figure 1.

### 2.2. Examination

#### 2.2.1. Anthropometric Measurements

Body measurements were measured using the bioelectrical impedance method [18]. Body composition (body weight, fat mass, and muscle mass) were measured using an Inbody 770 (Biospace, Seoul, Korea) from Biospace. Body fat mass and muscle mass were each divided by body weight and converted into percentages. For an accurate examination, the hands and feet were washed with alcohol to remove sweat, and both arms and legs were abducted and measured at 30 degrees so that the armpits and the groin did not touch each other. In addition, prior to the test, the subject fasted for 8 h and either controlled or measured actions such as high-intensity exercise or sauna use, which may cause water loss. The training was conducted during the winter season (January to February), when the athletes were not in season, so body composition analysis was not affected by in-season training or playing.

#### 2.2.2. Isokinetic Strength Test

Isokinetic muscle function was measured using a CSMi dynamometer and HUMAC software (CSMi HUMAC NORM, Stoughton, MA, USA, 2015). To increase the reliability and validity of accurate measurements and tests, the laboratory temperature was maintained at 20 °C to 25 °C, and there was no excessive eating or high-intensity exercise before the test. The extension and flexion of the knee joint were measured, an angular velocity of 60 degrees was set to measure muscle strength, and an angular velocity of 240 degrees was set to measure muscle endurance. Subjects were seated and the knee axis was aligned with the lateral epicondyle of the femur. The ankle pad was fixed to fit the distal part of the tibia. In addition, it was fixed using a pad so that the thigh and upper body did not move. The test subjects performed knee extension and flexion with concentric contraction. Sufficient explanation was given to help understand the test, and practice before the test was conducted. To induce familiarity with the test, the exercises were performed with submaximal muscle contraction 3 times at an angular velocity of 60 degrees and 3 times at a high speed at 240 degrees. The test range was set to 0–90 degrees, and the starting posture was measured at the 90-degree knee posture with the examiner’s signal first, followed by flexion with the next signal. After the practice, the actual test was measured 4 times at 60°/s, and the muscle endurance was measured by repeating it 25 times at 240°/s. Muscle strength was measured based on peak torque, and the unit was a newton meter (Nm), and an absolute value and a relative value of torque per body weight were used. Muscular endurance was measured based on the total amount of work performed 25 times at an angular velocity of 240 degrees with joules as the units, and absolute and relative values were used in the same way. If there was a past injury or pain in the knee, the healthy knee was examined first. After measuring both sides, the average values of both sides were used for analysis.

#### 2.2.3. Wingate Test

The Wingate test is a method designed to measure the anaerobic power for the load (kp; 1 kp = 50 watt) multiplied by 0.075 by weight, as suggested by Bar-Or [19]. Kp is a unit of force, and 1 kp is the magnitude of gravity for an object with a mass of 1 kg. In addition, the Wingate anaerobic test is the most commonly used method for testing the anaerobic power of athletes [20]. The Wingate test was performed using a cycle ergometer (Monark model 864 Crescent AB, Varberg, Sweden). The seat height was adjusted so that when the pedal was at the 6 o’clock position, the knee angles were 25 to 35 degrees [21,22].

The Wingate test includes 3 to 5 min of warm-up and exercise, with 80 revolutions per minute (RPM) under a light load of 50 watts (1 kp). When the participant’s preparation is complete, the examiner gives a “start” signal, and the calculated load (weight × 0.075; kp) is applied at the same time. The subject tries to exert the maximum RPM for 30 s, the inspector records revolutions per minute (RPM) every 5 s, and the highest RPM, minimum RPM, and average RPM are recorded at the end of the test. The maximum power was calculated throughout the test using the following formula.


Peak Power = Peak RPM/12 × kp × 6/0.083/6.12; (unit: watt)

In addition, the peak power was corrected to calculate the peak power per body weight (Peak Power/weight; watt) and the fatigue index (peak RPM − Lowest RPM/peak RPM × 100; %) was measured. After one test, a 2-min rest period was given. During this time, the pedal did not stop and 80 RPM at 1 kp was maintained. The test was repeated and measured five times in total.

### 2.3. Training Programs

#### 2.3.1. High-Intensity Interval Training (HIIT)

HIIT is based on the training theory recommended by the American College of Sports Medicine (ACSM) [23], and this study used the same repeated Wingate efforts method used in Takei’s study [24]. The purpose of anaerobic training is to strengthen the ability to move quickly, start quickly, exercise in a short amount of time, and sustain the high-intensity exercise necessary for badminton games. Due to the nature of badminton, one rally (the game time for which the score is recorded) is about 12 s on average; training under an extreme load for 30 s is designed to improve performance, considering that a rally often lasts up to 30 s [25]. The exercise program in this study was carried out using a stationary bicycle. The athletes trained three times a week. The warm-up and cool-down periods involved cycling at a low intensity for 5–10 min and 40–50%, respectively. A warm-up was performed while applying a load of 1 kp and maintaining a speed of 80 RPM. When the heart rate exceeded 50% of the maximum heart rate, the load was lowered or the RPM was adjusted to lower the heart rate. The total exercise period was 30 min. The exercise portion included a total of 10 bouts and was performed with an intensity of 90% heart rate maximum (HRmax) or higher for 30 s per 1 bout. The break time given per bout was 150 s, but during this break, exercise was maintained at a 50% HRmax level. The heart rate (Polar RS400, USA) was continuously checked, and if the subject’s heart rate dropped or the participant was tired, they were verbally encouraged. To improve anaerobic power, training was conducted to operate the pedal as quickly as possible at a given load for 30 s at 1 bout. Details on training are shown in Table 1.

#### 2.3.2. Moderate Continuous Training (MCT)

MCT refers to a general training method for muscle strength and endurance and consists of an exercise program with a total of 12 bouts. According to the training method recommended by ACSM, it includes warm-up exercises for 5–10 min, the main exercise for 30 min, and a cool-down exercise for 5–10 min [23]. The MCT group completed the same warm-ups as the HIIT group. In the main exercise section, the upper and lower body were trained with 6 types of exercises each. Weight training for muscle strength was set to 80–85% of 1 repetition maximum (RM) and 3 sets of 10 were performed. Exercise for muscular endurance was 1 RM. Participants trained in 3 sets of 25 reps by setting a 50–60% intensity of 1 RM. The equipment used in the main exercise session used 6 types of lower body exercise machines (leg extension, leg curl, leg press, inner thigh, hip abduction, and total hip extension) and upper body machines (abdominal, rotary torso, shoulder press, and chest press, lat-pulldown, and long pull). One bout consisted of 3 sets, and each set was performed 10 times to increase muscle strength and 25 times to increase endurance. The rest time was 30 s per set and 1 min per bout. During the total 4-week training period, in the 1st and 3rd weeks, the focus was on increasing muscle strength, and in the 2nd and 4th weeks, the focus was on increasing muscle endurance. Details on training are shown in Table 2.

#### 2.3.3. Data Analysis

Data were analyzed using SPSS 25.0 (IBM SPSS Ltd., Chicago, IL, USA) and were recorded as mean and standard deviation. The nonparametric method was applied, and the paired *t*-test was performed using the Wilcoxon method for the before and after comparison by means of repeated intra-group measurements, and the independent *t*-test was performed using the Mann–Whitney method for comparisons between the two groups. Repeated two-way ANOVA was performed to analyze the differences between time and group and before and after training. The significance level was set to *p* < 0.05.

## 3. Results

### 3.1. Participants’ Characteristics

Table 3 describes the general characteristics of the participants. There was no significant difference between the groups in the age, height, weight, and body mass index (BMI) values of the HIIT group and the MCT group.

### 3.2. Body Fat and Muscle Changes before and after Training

Table 4 shows the changes in fat and muscle after the 4-week training in the HIIT group and the MCT group. There was no statistical difference between groups before training in all items. In the HIIT group, body fat and body fat percentage decreased statistically significantly after training. There was also a significant decrease between groups. However, there was no statistical significance in terms of the muscle mass and muscle ratio after training in either group. In the MCT group, there was no statistically significant difference in any items before and after training.

### 3.3. Change in Anaerobic Power

Table 5 shows the results of anaerobic power, determined through the Wingate test. A total of five sets of Wingate tests were performed, and peak power and fatigue index were displayed according to the weight of each set. As a result, in the case of peak power, there was no statistical difference between the anaerobic power test and the fatigue index test performed before training in both the HIIT group and the MCT group. After 4 weeks of training, there was a statistically significant improvement in peak power and fatigue index in sets 1 and 2 in both groups. However, in the case of the HIIT group, statistically significant improvements in peak power and fatigue index were observed in the anaerobic power tests of three and four sets. In the case of the MCT group, the peak power and fatigue index were not different from the pre-test values in the 3rd and 4th sets. In the case of the 5th set, the peak power did not change in both groups, but the fatigue index had a statistically significant effect in the HIIT group.

### 3.4. Heart Rate Change after Anaerobic Power Test

Table 6 shows the pre-post results in regard to heart rate after the Wingate test. The heart rate at 1 min after the end of the test was subtracted from the maximum heart rate found through each set, and the value was divided by the maximum heart rate and expressed as a percentage. There was no statistical change in either group.

### 3.5. Isokinetic Muscle Function

Table 7 shows the pre-post results for the isokinetic muscle function test. In both the HIIT and MCT groups, there was no statistically significant difference between the groups in the pre-test period before training at an angular velocity of 60 degrees and an angular velocity of 240 degrees. After 4 weeks of training, there was a statistically significant increase in extension, extension/kg, flexion, and flexion/kg in both groups at a low speed of 60 degrees. However, there was no difference between the groups. However, in the high-speed test at 240 degrees, there was a statistically significant change before and after testing only in the HIIT group.

## 4. Discussion

There are several studies that have applied HIIT to badminton players. In a study conducted by Wee, 18 college badminton players underwent HIIT training for 4 weeks; their improvement in mean power was statistically significant in relation to VO2 max and anaerobic power ability [26]. Samsir reported a statistically significant increase in VO2 max and a 20-m shuttle run test, which is a comprehensive test of anaerobic ability, agility, and muscular endurance after HIIT for 10 weeks in 16 male adolescent badminton players [27]. Badminton requires better stamina than other sports [28]; based on this, this study also examined badminton players, and HIIT showed a statistically significant improvement in terms of body fat, anaerobic power, and isokinetic muscle endurance compared to MCT.

There are many studies that relate to the training period. In the case of short-term training, an improvement in physical strength and performance was reported even after 4–6 weeks of training [29]. In addition, Pritchard’s results showed that after the general population used HIIT 3 days a week for 4 weeks, the isometric strength of the lower body when using a leg extension machine, the isometric strength of the upper body when using a chest press machine, and standing jump performance increased statistically significantly [30]. In this study, the body fat, anaerobic ability, fatigue, and muscle endurance among the isokinetic muscle functions of the HIIT group improved in only a short period of 4 weeks. For the MCT group, there was no change in body composition, but the first two sets showed a significant increase in anaerobic power and a statistically significant decrease in muscle fatigue from the pre-test period. In addition, knee extension muscle strength and muscle endurance using isokinetic equipment showed a statistically significant increase in both HIIT and MCT groups compared to the pre-test period. Regarding knee flexion, the HIIT group showed a statistically significant increase in both muscle strength and muscle endurance. In the MCT group, there was a statistically significant increase in muscle strength, but the improvement in muscle endurance was not statistically significant.

Four weeks of HIIT proved to be an important factor in demonstrating the effectiveness of short-term training in fat and muscle changes in athletes. However, opinions are divided on the clear training effect of HIIT for youth. After reviewing the effect of short-term HIIT training as reported in 13 studies, Eddolls observed that six studies reported statistically significant changes in body mass index (BMI), %Fat, and fat-free mass (FFM) [31]. However, there was no consensus even on this result. Eddolls’s review showed some studies that reported increased BMI, and others that reported increased FFM. In this study, after training, the HIIT group showed a statistically significant loss of body fat. This should highlight how HIIT is based on aerobic exercise, whereas MCT is based on unilateral strength training and therefore the principle of training specificity [32]. Schubert reported that the resting metabolic rate (RMR) and VO2 max increased statistically significantly and body fat decreased due to Wingate training in 30 men and women who were not athletes over 4 weeks [33]. Therefore, it has been reported that Wingate-based training can have a significant beneficia; effect within a four-week training period.

There have been several prior studies on anaerobic power. According to Forster, the peak power output increased by 18% as a result of HIIT training for 55 healthy college students conducted three times a week for 8 weeks [34]. In this study, anaerobic power was increased in both HIIT and MCT groups even during the short training period of 4 weeks. A total of five sets of anaerobic power were tested. In particular, the HIIT group showed an increase of 12.4% in the first set, which was statistically significant up to the fourth set. In the case of the MCT group, there was a statistically significant increase in sets 1 and 2 compared to the pre-test period, but not in sets 3–5. As the set was repeated, the anaerobic power and muscular endurance of badminton players decreased, but the HIIT group was able to maintain the expression of power after 4 weeks of training, which is expected to improve performance. Similarly, muscle fatigue can be predicted based on the correlation between the highest and lowest RPMs during the test. In this area, the HIIT group showed a statistically significant decrease in fatigue compared to the MCT group. In the MCT group, after 4 weeks of training, there was a statistically significant decrease in the 1st and 2nd sets, but there was no significant difference from the pre-test period in the 3rd and 5th sets. Anaerobic power means instantaneous explosive power, and fatigue refers to how well it can be maintained at the highest RPM. Badminton rallies last up to 30 s, so fatigue and muscular endurance are important for players.

After the anaerobic power test, the heart rate indicates the body’s physiological response. In this study, there was no statistically significant change after training in both HIIT and MCT groups. Ramos et al. studied the effect of HIIT and MCT training on vascular function using a meta-analysis method and concluded that HIIT had a significant effect of 2.26% (*p* < 0.05) on vascular function, such as brachial artery flow-mediated dilation (FMD), compared to MCT, which in turn had a positive effect on biomarkers related to cardiorespiratory fitness and vascular function [35]. The ability to recover heart rate after an extreme anaerobic power test is also included in vascular function. However, there was no change in heart rate after training in this study. It may be that the 4-week training period was insufficient to show functional changes in heart rate. Furthermore, all of the variables that had an effect in Ramos’ study involved training for at least 12 weeks. Therefore, the variables related to the heart are not likely to change with only four weeks of training. Therefore, longer-term training is necessary in future studies.

The muscle strength and endurance in this study were measured using an isokinetic device. High-intensity interval training improves the molecular signaling pathways that control changes in muscles caused by endurance training, such as mitochondrial production and the ability to transport and oxidize carbohydrates and fats [36]. In this study, an angular velocity of 60°/s and an angular velocity of 240°/s were measured, and the measurement items were measured strength and endurance, respectively. As a result of 4-week training, there was a statistically significant improvement in muscle strength in both the HIIT and MCT groups, and no improvement in muscle endurance was observed in the MCT group. There was no statistical difference between groups in terms of muscle strength, but the HIIT group showed more improvement than the MCT group. In muscle endurance, the HIIT group showed a statistically significant difference, whereas the MCT group did not. This implies that adaptation due to training is possible in just 4 weeks.

This study has several limitations. First, the effectiveness of training was not identified by further subdividing the age of the adolescents. Therefore, it is necessary to investigate the effect of training on badminton players in middle and high school students separately. Second, it was not possible to control for food intake or daily life activities, which may have affected the results. Third, it was not possible to conduct additional types of training and comparative analysis to determine the effect of HIIT. Therefore, further research is needed to improve these limitations.

## 5. Conclusions

The HIIT group showed decreased body fat, improved anaerobic power, improved fatigue index, knee extension and flexion muscle endurance. Therefore, it is valuable to apply a short training period of HIIT to improve performance in youth badminton players.

## Figures and Tables

**Table 1:** Specific program of High-Intensity Interval Training (HIIT).

Frequency and Duration	Time		Program	Intensity
3 times/week For 4 weeks Training	5~10 min		Warm-up	40~50% HRmax, 50 RPM
	30 min/day	30 s	1st bout	Beyond 90% HRmax, Maximal Effort
		150 s	rest	Maintain 50% HRmax, 80 RPM
		30 s	2nd bout	Beyond 90% HRmax, Maximal Effort
		150 s	rest	Maintain 50% HRmax, 80 RPM
		30 s	3th bout	Beyond 90% HRmax, Maximal Effort
		150 s	rest	Maintain 50% HRmax, 80 RPM
		30 s	4th bout	Beyond 90% HRmax, Maximal Effort
		150 s	rest	Maintain 50% HRmax, 80 RPM
		30 s	5th bout	Beyond 90% HRmax, Maximal Effort
		150 s	rest	Maintain 50% HRmax, 80 RPM
		30 s	6th bout	Beyond 90% HRmax, Maximal Effort
		150 s	rest	Maintain 50% HRmax, 80 RPM
		30 s	7th bout	Beyond 90% HRmax, Maximal Effort
		150 s	rest	Maintain 50% HRmax, 80 RPM
		30 s	8th bout	Beyond 90% HRmax, Maximal Effort
		150 s	rest	Maintain 50% HRmax, 80 RPM
		30 s	9th bout	Beyond 90% HRmax, Maximal Effort
		150 s	rest	Maintain 50% HRmax, 80 RPM
		30 s	10th bout	Beyond 90% HRmax, Maximal Effort
		150 s	rest	Maintain 50% HRmax, 80 RPM
	5~10 min		Clean-up	40~50% HRmax, 50 RPM

Abbreviation: RPM, revolutions per minute; HRmax, heart rate maximum.

**Table 2:** Specific programs of Moderate Continuous Training (MCT).

Frequency and Duration	Time and Method			Program	Intensity
**3 times/week ** **For 4 weeks Training**	5~10 min			Warm-up	Cycle, 50 watt, 60~70 RPM
	30 min	Upper Extremity, 6 bouts, Lower Extremity, 6 bouts Total: 12 bouts 10 repetitions × 3 sets (for strength), 25 repetition × 3 sets (for endurance) Rest per set: 30 s Rest per bout: 60 s	Lower Extremity	Leg Extension	10 repetitions × 3 sets (for strength), 1 RM 80%~85%, 25 repetition × 3 sets (for endurance) 1 RM 50%~60% 1st, 3rd weeks: Strength training 2nd, 4th weeks: Endurance training
				Leg Curl	
				Leg Press	
				Inner Thigh	
				Outer Thigh	
				Total Hip Extension	
			Upper Extremity	Abdominal	
				Rotary Torso	
				Shoulder Press	
				Chest Press	
				Lat-Pulldown	
				Back Extension	
	5~10 min			Clean-up	Cycle, 50 watt, 60~70 RPM

RPM, revolutions per minute; HR, heart rate; RM, repetition maximum.

**Table 3:** General characteristics of participants.

Variables	HIIT	MCT	*p*
Age, years	16.3 ± 1.2	16.5 ± 1.0	0.213
Height, cm	174.9 ± 3.5	175.7 ± 3.1	0.512
Weight, kg	65.1 ± 6.8	66.0 ± 5.4	0.631
BMI, kg/m^2^	21.5 ± 1.8	21.7 ± 1.5	0.418

HIIT, high-intensity interval training; MCT, moderate continuous training, BMI, body mass index.

**Table 4:** Changes in body composition between groups according to training.

Variables	Group	Pre	Post	Diff (%)	Pre–Post, *p*	Time * Group, *p*
Fat, kg	HIIT	7.2 ± 2.2	6.6 ± 2.2	−9.1	0.016*	0.019 *
	MCT	7.4 ± 1.9	6.9 ± 1.7	−7.2	0.221	
	*p*	0.125	0.021 *			
Fat, %	HIIT	11.1 ± 2.6	10.2 ± 2.6	−7.8	0.022 *	0.028 *
	MCT	11.2 ± 2.7	10.5 ± 2.4	−6.8	0.191	
	*p*	0.214	0.043 *			
Muscle, kg	HIIT	30.8 ± 3.2	31.4 ± 3.4	1.9	0.121	0.415
	MCT	31.2 ± 2.8	31.6 ± 2.7	1.3	0.164	
	*p*	0.119	0.213			
Muscle, %	HIIT	47.3 ± 1.4	48.8 ± 2.0	3.1	0.144	0.612
	MCT	47.2 ± 1.5	48.1 ± 1.4	1.7	0.109	
	*p*	0.258	0.121			

* *p* < 0.05; HIIT, high-intensity interval training; MCT, moderate continuous training; Diff, different between pre- and post-training.

**Table 5:** Anaerobic power testing with the Wingate ergometer test.

Variables	Group	Pre	Post	Diff (%)	Pre–Post, *p*	Time * Group, *p*
Peak Power (P.P./BW)						
1	HIIT	11.3 ± 1.4	12.9 ± 1.8	12.4	0.003 *	0.314
	MCT	11.6 ± 1.4	12.4 ± 1.2	6.5	0.004 *	
	*p*	0.524	0.129			
2	HIIT	11.2 ± 1.5	12.3 ± 1.5	8.9	0.007 *	0.546
	MCT	11.4 ± 2.3	12.1 ± 1.1	5.8	0.019 *	
	*p*	0.268	0.211			
3	HIIT	10.9 ± 1.7	11.8 ± 1.4	6.8	0.029 *	0.019 *
	MCT	10.8 ± 1.6	11.4 ± 1.0	5.3	0.121	
	*p*	0.645	0.014 *			
4	HIIT	10.1 ± 1.2	11.0 ± 1.9	7.3	0.012 *	0.021 *
	MCT	10.2 ± 1.4	10.5 ± 2.1	2.9	0.062	
	*p*	0.341	0.011 *			
5	HIIT	9.3 ± 1.8	9.5 ± 1.7	2.1	0.445	0.129
	MCT	9.1 ± 1.6	9.3 ± 4.8	2.2	0.741	
	*p*	0.412	0.064			
Fatigue Index (F.I.)						
1	HIIT	32.4 ± 9.9	26.8 ± 10.6	−20.9	<0.001 *	0.841
	MCT	35.2 ± 10.9	28.5 ± 12.5	−23.5	0.003 *	
	*p*	0.512	0.721			
2	HIIT	40.3 ± 9.6	31.3 ± 10.1	−28.8	0.003 *	0.743
	MCT	42.7 ± 10.5	34.3 ± 14.9	−24.5	0.014 *	
	*p*	0.218	0.119			
3	HIIT	45.5 ± 9.2	39.0 ± 10.8	−16.7	0.029 *	0.032 *
	MCT	47.5 ± 11.3	45.0 ± 13.4	−5.6	0.121	
	*p*	0.347	0.024 *			
4	HIIT	49.4 ± 14.2	42.7 ± 12.5	−15.7	0.012 *	0.017 *
	MCT	50.0 ± 9.3	47.4 ± 13.9	−5.5	0.062	
	*p*	0.419	0.022 *			
5	HIIT	54.0 ± 15.0	51.0 ± 11.8	−5.9	0.045 *	0.003 *
	MCT	55.9 ± 12.9	54.4 ± 16.8	−2.8	0.741	
	*p*	0.612	0.019 *			

* *p* < 0.05; HIIT, high-intensity interval training; MCT, moderate continuous training; Diff, different between pre- and post-testing; P.P., peak power; BW, body weight; F.I., fatigue index.

**Table 6:** Heart rate after the Wingate test.

Set	Group	Pre	Post	Diff (%)	Pre–Post, *p*	Time * Group, *p*
1	HIIT	39.3 ± 4.5	42.0 ± 3.1	6.4	0.412	0.211
	MCT	41.0 ± 3.6	43.4 ± 4.1	5.5	0.319	
	*p*	0.514	0.748			
2	HIIT	35.3 ± 6.7	38.2 ± 4.5	7.6	0.424	0.342
	MCT	37.5 ± 2.5	41.8 ± 5.8	10.3	0.511	
	*p*	0.126	0.419			
3	HIIT	30.2 ± 5.1	33.0 ± 7.4	8.5	0.671	0.417
	MCT	32.5 ± 4.8	31.2 ± 4.7	−4.2	0.546	
	*p*	0.513	0.641			
4	HIIT	20.2 ± 6.6	24.2 ± 4.3	16.5	0.541	0.784
	MCT	19.9 ± 6.9	23.4 ± 5.9	15.0	0.097	
	*p*	0.663	0.518			
5	HIIT	19.3 ± 7.2	19.6 ± 5.8	1.5	0.417	0.646
	MCT	18.6 ± 8.5	21.6 ± 6.9	13.9	0.211	
	*p*	0.248	0.820			

Heart rate = ((max − recovery 1 min)/max) × 100; HIIT, high-intensity interval training; MCT, moderate continuous training; Diff, different between pre- and post-testing.

**Table 7:** Isokinetic strength test.

Variables	Group	Pre	Post	Diff (%)	Pre–Post, *p*	Time * Group, *p*
60°/s, Ext, Nm	HIIT	194.9 ± 30.4	223.4 ± 31.6	12.8	0.010 *	0.122
	MCT	201.9 ± 33.7	212.5 ± 28.3	5.0	0.015 *	
	*p*	0.121	0.221			
60°/s, Ext, Nm/kg	HIIT	2.99 ± 0.31	3.43 ± 0.31	13.1	0.011 *	0.746
	MCT	3.05 ± 0.42	3.22 ± 0.36	5.0	0.003 *	
	*p*	0.515	0.153			
60°/s, Flx, Nm	HIIT	117.8 ± 17.2	132.2 ± 13.9	10.9	0.002 *	0.879
	MCT	122.6 ± 24.5	129.2 ± 17.7	5.1	0.004 *	
	*p*	0.247	0.412			
60°/s, Flx, Nm/kg	HIIT	1.81 ± 0.26	2.03 ± 0.26	11.3	0.045 *	0.412
	MCT	1.86 ± 0.31	1.96 ± 0.21	5.1	0.014 *	
	*p*	0.416	0.163			
240°/s, Ext, total joule	HIIT	2690.4 ± 540.9	2900.9 ± 561.7	7.2	0.003 *	0.035 *
	MCT	2609.4 ± 447.1	2763.9 ± 456.5	5.6	0.015 *	
	*p*	0.258	0.011 *			
240°/s, Ext, Total Joule/kg	HIIT	41.32 ± 6.04	44.56 ± 5.81	7.3	0.005 *	0.002 *
	MCT	39.53 ± 6.60	41.86 ± 7.12	5.5	0.129	
	*p*	0.426	0.035 *			
240°/s, Flx, total Joule	HIIT	1576.7 ± 281.5	1899.8 ± 318.4	17.0	0.006 *	0.004 *
	MCT	1608.8 ± 336.2	1767.8 ± 309.7	9.0	0.217	
	*p*	0.416	0.003 *			
240°/s, Flx, total Joule/kg	HIIT	24.21 ± 3.94	29.17 ± 3.33	17.0	0.002 *	0.005 *
	MCT	24.37 ± 5.19	26.82 ± 4.34	9.1	0.059	
	*p*	0.641	0.012 *			

* *p* < 0.05; HIIT, high-intensity interval training; MCT, moderate continuous training; Diff, different between pre- and post-testing.

## Data Availability

The data are not publicly available due to privacy or ethical reasons.
